# Outcomes of Primary Repair and Anastomosis for Traumatic Colonic Injuries in a Tertiary Trauma Center

**DOI:** 10.3390/medicina56090440

**Published:** 2020-08-31

**Authors:** Marie Shella De Robles, Christopher J. Young

**Affiliations:** 1 Department of Colorectal Surgery, Royal Prince Alfred Hospital, Sydney 2050, New South Wales, Australia; shella.derobles@gmail.com; 2 Discipline of Surgery, The University of Sydney, Sydney 2050, New South Wales, Australia

**Keywords:** colonic injury, blunt trauma, penetrating trauma, resection, primary repair, colostomy

## Abstract

*Background*: Surgical management for traumatic colonic injuries has undergone major changes in the past decades. Despite the increasing confidence in primary repair for both penetrating colonic injury (PCI) and blunt colonic injury (BCI), there are authors still advocating for a colostomy particularly for BCI. This study aims to describe the surgical management of colonic injuries in a level 1 metropolitan trauma center and compare patient outcomes between PCI and BCI. *Methods*: Twenty-one patients who underwent trauma laparotomy for traumatic colonic injuries between January 2011 and December 2018 were retrospectively reviewed. *Results*: BCI accounted for 67% and PCI for 33% of traumatic colonic injuries. The transverse colon was the most commonly injured part of the colon (43%), followed by the sigmoid colon (33%). Primary repair (52%) followed by resection-anastomosis (38%) remain the most common procedures performed regardless of the injury mechanism. Only two (10%) patients required a colostomy. There was no significant difference comparing patients who underwent primary repair, resection-anastomosis and colostomy formation in terms of complication rates (55% vs. 50% vs. 50%, *p* = 0.979) and length of hospital stay (21 vs. 21 vs. 19 days, *p* = 0.991). *Conclusions*: Regardless of the injury mechanism, either primary repair or resection and anastomosis is a safe method in the management of the majority of traumatic colonic injuries.

## 1. Introduction

**Figure 1:**
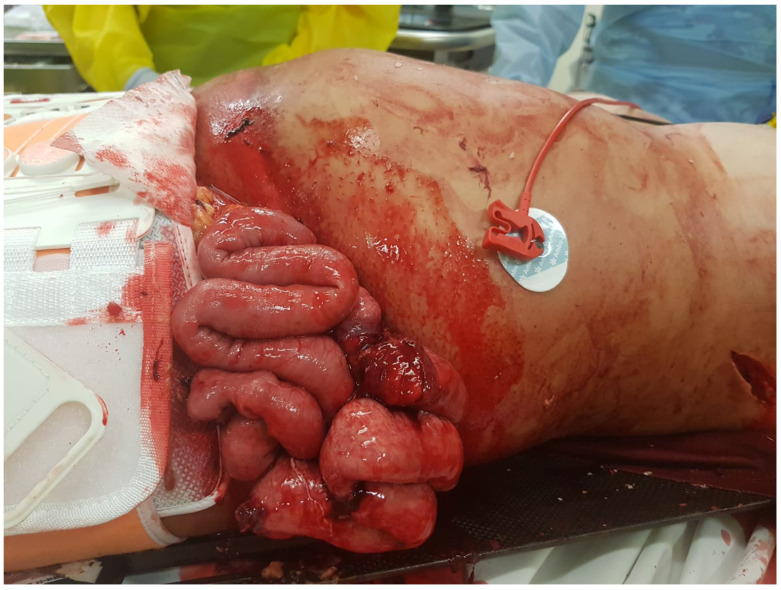
Bowel evisceration secondary to blunt trauma from a motor vehicle accident.

Major abdominal trauma is commonly encountered as a polytrauma, resulting in imminently life-threatening condition. It is particularly challenging to diagnose and manage due to the presence of distracting injuries and altered mental status of the patients. Penetrating injuries are easier to detect. A hemodynamically stable patient often complains of abdominal tenderness, and their examination can reveal peritoneal signs. Blunt abdominal trauma injuries are notoriously harder to detect with patients often presenting with generalized abdominal tenderness. No diagnostic test or combination of clinical findings can reliably exclude blunt abdominal trauma. Blunt colonic injuries (BCIs) can be very destructive (Figure 1), and are associated with multiple organ damage causing significant clinical compromise [1]. Even with relatively prompt surgery, these patients are at significantly higher risk of severe complications and increased length of stay. A delay in operative intervention is associated with even more serious morbidity [1,2,3].

The incidence of abdominal complications after traumatic colonic injuries is very high, with the sepsis rate as high as 20% [4]. Various factors including mechanism of injury, grade of colonic injury and method of repair used have all been suggested as possible risk factors for colon-related complications [4]. There are very few studies with adequate sample size and design that provide clinically useful conclusions on the management of BCI. Therefore, it is not evident whether the principles that guide the management of penetrating colonic injury (PCI) should be applied to these patients.

This study aims to describe the surgical management of traumatic colonic injuries in our institution and to compare patient outcomes between BCI and PCI.

## 2. Materials and Methods

A retrospective review of the trauma registry data at Royal Prince Alfred Hospital (RPAH) was performed. RPAH is a major trauma center situated in the inner city of Sydney, Australia. The hospital treats approximately 270 major trauma cases a year and over 3000 minor trauma cases.

Patients 15 years and older who underwent laparotomy for the management of traumatic colonic injury between the 1 January 2011 and 31 December 2018 were included in this study. Patient demographics, mechanisms of injury, treatment course and complications were reviewed from individual patients’ medical records. Diagnoses of injuries were confirmed either radiologically (via the radiologists’ reports of computed tomography scans) or operatively (via the operation report documented by the surgeon). The approach to management was dependent on the operating surgeon, their preference and the extent of the injury. Shock class at presentation was based on the Advanced Trauma Life Support (ATLS) definition. Colonic injuries were categorized using the American Association for the Surgery of Trauma (AAST) injury scoring system. 

Statistical analysis was completed using SPSS version 23.0 software (IBM, Armonk, New York, NY, USA). Categorical variables were compared using the chi-square test, and continuous variables were compared using an independent-sample t-test. *p* < 0.05 was considered statistically significant. 

**Ethics:** Ethical approval (protocol no X16-0207) was obtained from the Sydney South West Area Health Service Ethics Review Committee (RPAH Zone).

## 3. Results

The study group consisted of 21 patients. This comprised 16% (21/131) of all trauma laparotomies performed at RPAH from January 2011 to December 2018. The patient characteristics are shown in Table 1. The majority (81%) of the patients were male with a mean age of 39.7 years (range 18–92). Two-thirds of the injuries were secondary to blunt trauma, and the rest were from penetrating anterior abdominal stab wounds. Perioperative intravenous antibiotics were used in all patients. More than half of the patients (62%) required admission to intensive care unit postoperatively.

In the absence of physical findings that warrant an urgent need for exploratory laparotomy, either a focused assessment sonography for trauma (FAST) or computerized tomography (CT) can was used as adjuncts to help identify, prioritize, and guide the management of other injuries. A FAST was performed for all of the patients while an abdominopelvic CT Scan was done for 76% of the patients.

The transverse colon was the most commonly injured part of the colon, followed by the sigmoid colon. The majority of the patients (81%) had at least one other associated intra-abdominal organ injury. The most commonly involved were the small bowel (n = 12), liver (n = 5), pancreas (n = 2), kidney (n = 1), spleen (n = 1), and stomach (n = 1). Aside from concomitant intra-abdominal injuries, more than half (57%) of the patients also had associated extra-abdominal injuries requiring orthopaedic (n = 6), vascular (n = 5), cardiothoracic (n = 5) and neurosurgical (n = 5) consults. Concomitant extra-abdominal injuries were significantly more common in blunt trauma (*p* = 0.016).

Table 2 compares clinical outcomes between BCI and PCI. Regardless of the mechanism of injury, there was no difference regarding the colon injury grade (*p* = 0.163), shock class at presentation (*p* = 0.392) and method of repair used (*p* = 0.372). Primary repair of colon injuries (52%) was the most common operation performed, which was followed by resection and primary anastomosis (38%) and proximal diversion (10%). There was no difference in the method of repair used in both groups (*p* = 0.372).

The mean length of hospital stay was 20.7 days (range 2–93). The overall complication rate was 52%, with lower respiratory tract infection (n = 4) being the most common followed by wound infection (n = 2), intra-abdominal collection (n = 1) and prolonged ileus (n = 1). There were no leaks from the anastomosis or primary repair sites. Three patients died post-operatively, attributed to the significant brain and thoracic injuries rather than to the management of the colonic injury itself. Postoperative complication rates and the length of hospital stay were not significantly different between the two groups (*p* = 0.183). 

There was no significant difference comparing patients who underwent primary repair, resection-anastomosis and colostomy formation in terms of complication rates (55% vs. 50% vs. 50%, *p* = 0.979) and the length of hospital stay (21 vs. 21 vs. 19 days, *p* = 0.991).

## 4. Discussion

Trauma surgery has evolved significantly throughout centuries, from the teachings of World War II when a colostomy was the standard method of addressing colonic injuries, to the current increasing confidence in primary repair or resection and anastomosis. In 1991, Chappuis et al. published the landmark paper on colonic injuries, describing primary repair as a safe method with the ability to be considered as the treatment of preference for nearly all penetrating colonic injuries, independent of associated risk factors. In this study, 28 patients who were treated with primary repair or resection and anastomosis were compared to 28 patients who were treated with diversion. There was no anastomotic leak, or mortality reported in the cohort. Additionally, the incidence of septic-related complications was similar in both groups (21.4% vs. 17.9%) [5].

Similar to PCI, the use of primary repair or resection with primary anastomosis has been suggested for BCI [1]. Primary repair avoids stoma complications and a second operation. It also minimizes postoperative complications, and reduces the overall financial strain [1]. Hence, it should be considered as an option whenever possible. As a general rule, the primary repair is not ideal for patients with shock, significant blood loss, two or more solid organ injuries, moderate to severe faecal contamination, an operative delay of at least eight hours, and destructive colonic and abdominal wall injuries requiring extensive resection [6]. These rules generally guide PCI but may also be used to guide management of BCI, where there is a paucity of literature on optimal management.

**Figure 2:**
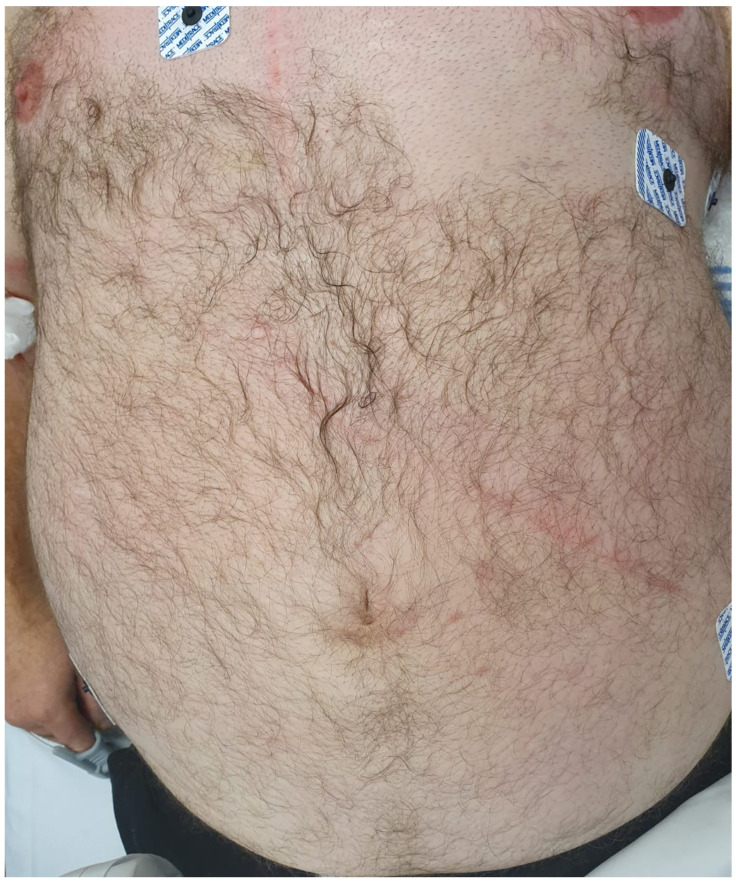
Seatbelt sign in a patient who sustained blunt abdominal trauma from a motor vehicle accident. The presence of a “seatbelt mark” should raise the index of suspicion for hollow viscus injury.

The overall reported incidence of BCI ranges from 0.1 to 13% [2,3,7,8,9]. BCI is usually associated with traffic trauma. The abrupt deceleration in such cases can cause significant mesenteric tears and colonic ischemia. Similarly, colonic injuries can also be incurred in situations where a transient closed loop results in either ischemic necrosis or blowout perforations. Seatbelt use is usually associated with an increased risk of hollow viscus injury [9] (Figure 2). Around 3% of the patients undergoing trauma laparotomy are noted to have destructive or full-thickness colonic perforations [9,10]. In some cases, colonic injuries may initially present subtly, either as colonic wall hematoma or contusion. These patients should be closely monitored because delayed perforation can still occur several days after the injury.

Blunt colonic injury is usually a diagnostic dilemma and associated with significant delay. Clinical assessment is often tricky and mostly unreliable due to the presence of distracting injuries and shock. Less than half of colonic blunt injuries have sufficient clinical findings to justify the need for an emergent laparotomy. Clinical assessment alone may result in negative exploratory laparotomy rates up to 40%, with associated morbidity ranging from 5 to 20% [1,11]. In our institution, a focused assessment with sonography for trauma (FAST) is generally used as the first step of the diagnostic studies. A positive FAST is defined as any free fluid in any of the four areas including the pericardium, hepatorenal recess, perisplenic space, and pelvis. This fluid, in the context of traumatic injury, usually means bleeding. Although FAST can easily be performed in the emergency department, the Eastern Association for the Surgery of Trauma (EAST) multi-institutional hollow viscus injury study reported only a 50% sensitivity and a 60% positive predictive value for FAST [3]. An abdominal CT Scan has also been recommended as an adjunct to help detect hollow viscus injuries. CT findings suggestive of a significant intra-abdominal injury were free intra-abdominal air or fluid, usually with concomitant solid organ injuries. However, the lack of these findings does not mean that no operative intervention is necessary. The reported sensitivity and specificity of a CT Scan in detecting blunt bowel or mesenteric injuries were 88% and 99%, respectively [8].

Delayed operative exploration of colonic injuries is recognized as an essential factor in the development of faecal contamination and is associated with a higher incidence of morbidity [12]. Severe faecal spillage is a significant independent risk factor for abdominal sepsis [4,9,13,14,15,16,17]. Most of the patients at our institution were operated on in the first 12 h, and most of them only had mild to moderate faecal contamination. Although delays in the operative management of colonic perforations increase the risk of septic complications, the length of delay over which the complication rate increases are not evident. Some studies suggest that this critical delay is six hours, while others extend it to 12 h [13,16,18]. It seems that the degree of contamination is much more critical than the operative delay and the time delay itself. Therefore, it should not be used as an absolute criterion that will dictate the method of repair. Recent large, prospective, observational, and randomized studies have shown that the method of colonic management does not influence the overall septic complication rate [4,5,19].

**Figure 3:**
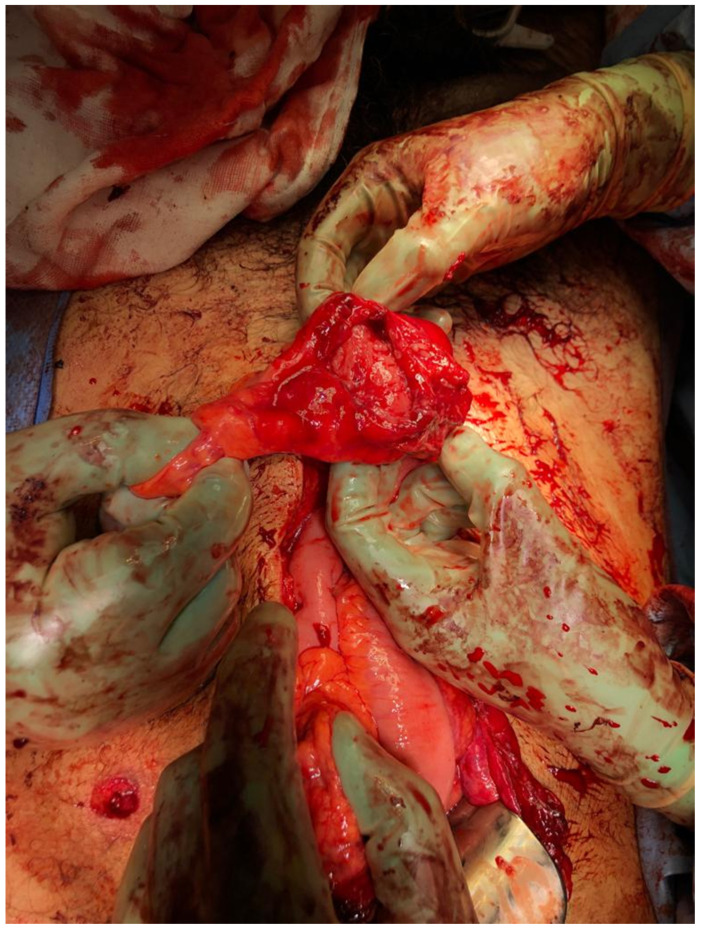
Transverse colonic injury secondary to blunt abdominal trauma.

Similar to other studies, the transverse colon was the most commonly injured segment of the colon, followed by the sigmoid colon [1,20]. The vulnerability is attributed primarily to its anatomical position (Figure 3). In BCI, the mechanism is usually due to the shearing forces and compression between the anterior abdominal wall and pelvis or lumbar spine [21]. The colon mesentery is also predisposed to laceration or avulsion-type injuries [21,22].

Traditionally, left-sided colonic injuries, particularly when associated with concomitant intra-abdominal injuries, were treated with resection and proximal end colostomy. There is no convincing evidence demonstrating significant difference in the post-operative complications when comparing right and left colonic injuries [4,11]. The cornerstone of successful colonic anastomosis is good blood supply and therefore this should always be ensured when repairing colonic injuries.

A higher incidence of mortality has been suggested among patients with severe intra-abdominal injuries, mandating formation of colostomy rather than primary colonic repair or anastomosis. However, the current literature has shown that despite the presence of multiple intra-abdominal injuries, the management of colonic injury does not significantly affect the incidence of perioperative complications, particularly intra-abdominal sepsis [7,13,19]. A few studies even suggested that the formation of a diverting colostomy in these high-risk patients may contribute to a higher incidence of intra-abdominal sepsis [4].

## 5. Conclusions

We are encouraged by our results demonstrating no significant difference in morbidity or mortality rates, regardless of the mechanism of injury and method of repair used. Primary repair of colonic injuries for both BCI and PCI can be the first choice of management for patients with lower colonic injury grade, mild to moderate faecal contamination, and without shock. Similar to PCI, diversion is not mandatory for the treatment of BCI. Colonic diversion is usually associated with a worse quality of life and requires an additional operation for reversal. In BCI, primary repair or resection with anastomosis should be considered whenever possible. The limitations of this study are the retrospective design and the small number of patients included. Future prospective studies should evaluate patients with trauma scoring systems to provide a better and more comprehensive assessment of injuries and hemodynamic status at presentation. 

## Figures and Tables

**Table 1:** Demographics of patients undergoing laparotomy for traumatic colonic injury.

	n = 21
Age in years; mean + SD ^√^; range	39.7 + 20.0; 18–92
Sex	
Male	17 (81%)
Female	4 (19%)
ATLS ^√^ shock class	
1	8 (38%)
2	5 (24%)
3	5 (24%)
4	3 (14%)
Mechanism of injury	
Penetrating (stab) trauma	7 (33%)
Blunt trauma	14 (67%)
Location of injury	
Transverse colon	9 (43%)
Sigmoid colon	7 (33%)
Splenic flexure	2 (9.5%)
Hepatic flexure	1 (5.0%)
Ascending colon	2 (9.5%)
AAST ^√^ colonic injury grade	
1	17 (81%)
2	2 (9%)
3	1 (5%)
4	1 (5%)
Management	
Primary repair	11 (52%)
Resection-anastomosis	8 (38%)
Diversion	2 (10%)
Associated intra-abdominal injuries	17 (81%)
Associated extra-abdominal injuries	12 (57%)
Number of associated organ injury	
less than 2	13 (62%)
2 or more	4 (19%)
none	4 (19%)
Intraoperative blood transfusion	8 (38%)
Post-operative ICU admission	13 (62%)
Post-operative complication	11 (52%)

^√^ SD—Standard Deviation; ATLS—Advanced Trauma Life Support; AAST—American Association for the Surgery of Trauma.

**Table 2:** Comparing outcomes based on mechanism of injury.

	BCI (n = 14)	PCI (n = 7)	
Mean age in years	38.8	41.4	*p* = 0.824 ^
Sex (Male: Female)	5:02	12:02	*p* = 0.574 *
ATLS ^√^ shock class			
1	4	4	
2	3	2	*p* = 0.392
3	4	1	
4	3	0	
AASTÖ colonic injury grade			
1	12	5	
2	0	2	*p* = 0.163
3	1	0	
4	1	0	
Management			
Primary repair	6	5	
Resection-anastomosis	6	2	*p* = 0.372
Diversion	2	0	
Other intra-abdominal injuries	13	4	*p* = 0.088 *
Associated extra-abdominal injuries	11	1	*p* = 0.016 *
Number of associated organ injury			
less than 2	9	4	
2 or more	4	0	*p* = 0.076
none	1	3	
Post-operative ICU admission	11	2	*p* = 0.056 *
Post-operative complication	9	2	*p* = 0.183 *
Mean length of hospital stay in days	26.0	10.0	*p* = 0.088 ^

* Fisher’s Exact; ^ t-test. ^√^ ATLS—Advanced Trauma Life Support; AAST—American Association for the Surgery of Trauma.
